# Genomic Studies of Plant-Environment Interactions

**DOI:** 10.3390/ijms23115871

**Published:** 2022-05-24

**Authors:** Man-Wah Li, Hon-Ming Lam

**Affiliations:** School of Life Sciences and Centre for Soybean Research of the State Key Laboratory of Agrobiotechnology, The Chinese University of Hong Kong, Shatin, Hong Kong, China; limanwah@cuhk.edu.hk

Plants have been evolving for millions of years to survive in their fast-changing environments, by promoting beneficial interactions with other organisms or taking advantage of new conditions in the physical environment, while finding ways to repel pathogens and pests or tolerate unfavorable conditions. The ability of the plant to adapt to the environment is mainly encoded in its genome. On the other hand, organisms that interact with plants have also developed unique sets of genetic elements to ensure successful interactions. Recent advancements in genome biology have unveiled the underlying mechanisms of genetic and epigenetic regulations of plant-environment interactions. This Special Issue explores the latest conceptual and technological breakthroughs in this aspect.

Through photosynthesis, plants produce sugars in the leaf mesophyll cells. These sugars are required as building blocks and an energy source for various vital functions throughout the whole organism. Thus, efficient deployment of these photosynthates is required to maintain the plant’s health. Sugars Will Eventually be Exported Transporters (SWEETs) are a class of sugar transporters involved in biological processes such as reproduction, seed-filling, phloem-loading, plant-microbe interactions, and sugar-loading upon osmotic stresses [1,2]. It is interesting that SWEETs play opposite roles in response to pathogen infection, as some of them cater to the pathogen’s need for sugars upon induction by pathogen effectors, while others remove sugars from the site of infection to minimize loss [2,3]. Watermelon (*Citrullus lanatus* [Thunb.]) produces highly sugary fruits. Xuan et al. identified 22 SWEET-encoding genes in the *C. lanatus* genome (*ClaSWEET*) [4]. Expression studies demonstrated that the *ClaSWEETs* were responsive to *Fusarium oxysporum* infection, drought, salt, and low-temperature stress [4]. This study establishes the genetic foundation for future studies on the roles of *ClaSWEETs* in the interactions between the watermelon plant and its environment.

Serving as functional units in the cell, the spatial and temporal distribution/compartmentalization of specific proteins are critical for proper cellular functions and thus the survival of the entire organism. Cells possess different ways to clear themselves of undesirable proteins, one of which is the ubiquitin-proteasome system (UPS), crucial for the precise removal of designated proteins. Compared to the UPS in animals, the plant UPS has an extensive collection of E3 ligase-encoding genes in the genome, which explains the high degree of substrate specificity [5]. It is believed that the expansion of E3 ligases in plants was a measure to cope with environmental changes as plants are incapable of relocating themselves away from stresses [5]. The RING/U-box E3 ligases are involved in growth and development, metabolism, and stress responses, etc [5,6]. Kim et al. identified 73 homologous U-box E3 gene pairs in the allohexaploid genome of wheat, constituting 213 U-box E3 ligase-encoding genes in total [6]. Through analyzing the transcriptome, the expressions of the majority of U-box E3 ligase-encoding genes in wheat were found to be responsive to drought, heat and cold stress, implying their potential roles in abiotic stress responses.

Reactive oxygen species (ROS) are mainly produced as metabolic by-products in plants. In most cases, ROS are tightly tied to detrimental stress responses, but in reality, a controlled production of ROS could serve as a defense mechanism toward stresses [7]. The plant genome encodes a few different classes of ROS-scavenging enzymes. One of them is catalase, which can convert hydrogen peroxide (H_2_O_2_) into harmless water and oxygen. Raza et al. identified 14 catalase-encoding genes and six microRNAs (miRNAs) from two miRNA families that target three of the catalase genes in the rapeseed (*Brassica napus* L.) genome [8]. The *cis*-elements in the promoters of these catalase genes were predicted. Most of the 14 genes were responsive to at least one environmental stress such as cold, salinity, waterlogging, abscisic acid, and gibberellic acid treatments. This study provides important genomic information for further investigations into the roles of catalase genes in the interactions of *B. napus* with its environment.

Trading off seed dormancy in exchange for better germination has resulted in the undesirable pre-harvest sprouting (PHS) of cereal crops during domestication [9]. PHS is especially common in warm and highly humid regions, causing significant yield loss in cereal crops. Being able to suppress PHS without re-introducing seed dormancy has become a great challenge to crop improvement. Zhu et al. have identified an abscisic acid (ABA)-regulated basic leucine zipper-encoding gene from rice (*OsbZIP09*) that is involved in PHS [10]. They found that the mutation of *OsbZIP09* could partially suppress PHS with only minor delay in seed germination [10]. Through RNA-seq and DNA affinity purification sequencing (DAP-seq), the direct targets of OsbZIP09 were revealed [10].

Underneath the soil surface, there are enormous microbe communities that play essential roles in plant growth in both beneficial and detrimental ways. The diversity and composition of soil microbes can also serve as an indicator of the health of the plantation. Poplar trees are planted for soil restoration and landscape protection, and the timber is also an important raw material for industries. Liu et al. compared the soil microbe communities of three different plantations with different degrees of poplar tree degeneration using a metagenomic strategy, and unveiled the correlation between the health of poplar trees and the soil microbial diversity and functions [11].

Soil-borne pathogens are one of the threats compromising crop productivity. *Pythium* is a genus in Phylum Oomycota. A majority of species within this genus are pathogenic toward plants and animals, and different *Pythium* species have different host ranges. Mohammadi et al. isolated *Pythium brassicum* P1, which has a much narrower host range compared to other *Pythium* spp. [12]. Through whole-genome sequencing and *de novo* genome assembly, it was found that the *P. brassicum* P1 genome lacks the RxLR effectors or cutinase-encoding genes and has a lower number of genes encoding other pathogenic proteins than other *Pythium* species [12]. These features might have resulted in the low pathogenicity of *P. brassicum* P1. These discoveries may help reveal the molecular mechanisms behind host specificity of *Pythium* spp.

Cadmium (Cd) is toxic to both plants and humans. Excessive Cd would lead to defective plant growth and eventually affect crop yield. Unfortunately, some of the arable lands are polluted with Cd due to anthropogenic activities [13], and this directly affects food security and food safety. Understanding the plant responses toward Cd toxicity will be important for improving Cd tolerance in plants. Niekerk and colleagues have reviewed the current understanding on Cd stress responses in plants at the epigenetic level, a relatively unexplored area in plant heavy metal tolerance research [14]. The aspects covered in the review include chromatin remodelling, histone modification, DNA methylation, and microRNA in Cd toxicity response and tolerance in plants [14], providing a framework for future studies.

## Data Availability

Not applicable.
